# Effects of statin treatment in patients with coronary artery disease and chronic kidney disease

**DOI:** 10.1007/s00380-013-0325-2

**Published:** 2013-02-21

**Authors:** Hidehiro Kaneko, Junji Yajima, Yuji Oikawa, Shingo Tanaka, Daisuke Fukamachi, Shinya Suzuki, Koichi Sagara, Takayuki Otsuka, Shunsuke Matsuno, Ryuichi Funada, Hiroto Kano, Tokuhisa Uejima, Akira Koike, Kazuyuki Nagashima, Hajime Kirigaya, Hitoshi Sawada, Tadanori Aizawa, Takeshi Yamashita

**Affiliations:** Department of Cardiovascular Medicine, The Cardiovascular Institute, 3-2-19 Nishiazabu, Minato-ku, Tokyo, 106-0031 Japan

**Keywords:** Statin, Japanese, Chronic kidney disease, Coronary artery disease, Percutaneous coronary intervention

## Abstract

Statins reduce cardiovascular morbidity and mortality from coronary artery disease (CAD). However, the effects of statin therapy in patients with CAD and chronic kidney disease (CKD) remain unclear. Within a single hospital-based cohort in the Shinken Database 2004–2010 comprising all patients (*n* = 15,227) who visited the Cardiovascular Institute, we followed patients with CKD and CAD after percutaneous coronary intervention (PCI). A major adverse cardiovascular and cerebrovascular event (MACCE) was defined by composite end points, including death, myocardial infarction, cerebral infarction, cerebral hemorrhage, and target lesion revascularization. A total of 391 patients were included in this study (median follow-up time 905 ± 679 days). Of these, 209 patients used statins. Patients with statin therapy were younger than those without. Obesity and dyslipidemia were more common, and the glomerular filtration rate (GFR) was significantly higher, in patients undergoing statin treatment. MACCE and cardiac death tended to be less common, and all-cause death was significantly less common, in patients taking statins. Multivariate analysis showed that low estimated GFR, poor left ventricular ejection fraction, and the absence of statin therapy were independent predictors for all-cause death of CKD patients after PCI. Statin therapy was associated with reduced all-cause mortality in patients with CKD and CAD after PCI.

## Introduction

Chronic kidney disease (CKD) increases the risk of cardiovascular mortality and morbidity [1, 2]. Furthermore, patients with coronary artery disease (CAD) are believed to have more frequently impaired renal function, and reduced renal function may result in worse clinical outcomes after revascularization therapy [2–5]. Statins reduce cardiovascular morbidity and mortality from CAD as a primary [6–8] and secondary [9–13] preventative. In addition, aggressive statin treatment reduces future cardiovascular events, even in CKD patients [14, 15].

CAD is becoming more common in the elderly as the mean age of the population increases in Japan [16]. Moreover, we previously reported that cardiovascular events, including cardiac death, nonfatal myocardial infarction (MI), or readmission for heart failure, increase significantly with declining estimated glomerular filtration rate (eGFR) [17]. In Japanese patients with acute MI, early lipid-lowering treatment with statins decreases recurring cardiovascular events—in particular, congestive heart failure [18]. Intensive statin treatment appears to be more effective than standard statin doses after acute coronary syndrome [19, 20] and stable angina pectoris [21]. However, the standard statin doses used in previous studies conducted in Western countries were larger than the standard doses approved in Japan. Individuals of Asian descent are thought to be more responsive than Caucasians to therapeutic drugs, and the respective standard doses appear to provide the same benefit to Asian patients [22]. We previously reported that statin therapy initiated early after the diagnosis of CAD may decrease the risk of fatal events in Japanese CAD patients [23]; however, the impact of statin therapy in patients with CKD and CAD after percutaneous coronary intervention (PCI) was less well defined.

A prospective cohort study, the Shinken Database, was designed to investigate the morbidity and mortality of Japanese patients with CAD who underwent PCI. In this study, using this database we evaluated the effects of statin treatment in patients with CKD and CAD after PCI.

## Patients and methods

### Study patients and protocols

The Shinken Database comprises all new patients who visit the Cardiovascular Institute in Tokyo, Japan (“Shinken” is an abbreviated name in Japanese for the name of the hospital), and excludes patients with active cancer and foreign travelers. The principal aim of this hospital-based database is surveillance of the prevalence and prognosis of cardiovascular diseases in the urban areas of Japan [24]. The registry began in June 2004, and thereafter patients have continually been registered in the database on an annual basis. The data in the present study were derived from this database between June 2004 and March 2011 (Shinken Database 2004–2010), including 15,227 new patients. Those patients with CKD and CAD who underwent PCI (*n* = 391) were enrolled in this study. PCI procedures including stent selection were performed by experienced operators. The following data were obtained: age, gender, height, body weight, prior history of MI, PCI, and coronary artery bypass graft (CABG), coronary risk factors, laboratory data, types of the implanted stents (bare-metal stent and/or drug-eluting stent), and medications at primary PCI. Ultrasound cardiography was routinely performed at the time of PCI.

### Patient follow-up

The health status, incidence of cardiovascular events, and mortality are maintained in the database through linking with the medical records of the hospital, and prognostic study documents are sent annually to those who discontinued hospital visits or were referred to other hospitals.

In the present data analysis, data from after April 1, 2011 were excluded. The end of the follow-up period was therefore defined by: (1) the date of death, if the date was prior to March 31, 2011; (2) the final hospital visit or the final response to our prognostic study documents prior to March 31, 2011; or (3) March 31, 2011, when the date of death, the final hospital visit, or the final response to our study documents was later than April 1, 2011.

### Ethics

The ethical committee of the Cardiovascular Institute granted ethical permission for this study, and all patients provided written informed consent.

### Definitions

We confirmed the deaths of study patients in the medical records of our hospital or by the information obtained from follow-up. Body mass index (BMI) was calculated at initial PCI by dividing the patient’s measured weight (in kilograms) by the square of the height (in meters); obesity was defined as a BMI of ≥25 kg/m^2^. GFR was calculated using the GFR equation designed for the Japanese population: GFR = 194 × (serum creatinine)^−1.094^ × (age)^−0.287^ × (0.739, if female) [25]. CKD was defined as eGFR <60 ml/min/1.73 m^2^. Target lesion revascularization (TLR) is defined as any repeat revascularization procedure (percutaneous or surgical) of the original target lesion site, including the stented plus edge segments (typically 5 mm proximal and distal to the stent). A major adverse cardiovascular and cerebrovascular event (MACCE) was defined as a composite end point including all-cause death, MI, cerebral infarction, cerebral hemorrhage, and TLR.

### Statistical analysis

Categorical and consecutive data are presented as number (%) and mean ± standard deviation (SD), respectively. The unpaired *t* test was used for comparison of consecutive variables between the two groups. Chi-square analysis was used to compare categorical variables. Long-term event-free survival was estimated using Kaplan–Meier curves, and the log-rank test was used to assess the significance of differences between patients with and without statin treatment. Univariate Cox regression analysis was used to identify cofactors with significant effects on all-cause death in CKD and CAD patients after PCI. Multivariate Cox regression analysis was performed to determine the independent prognostic factors for all-cause death of CKD and CAD patients after PCI. A probability value of less than 0.05 was considered to indicate a statistically significant difference. These analyses were performed using SPSS software (SPSS, Chicago, IL, USA), version 19.0.

## Results

### Patients’ characteristics

Of 391 patients, 209 (54 %) were taking statins. The median follow-up period was 905 ± 679 days. Patients taking statins were younger than patients without statins (68.7 ± 10.1 vs 72.0 ± 9.9 years, *P* = 0.001). Obesity (43.3 % vs 28.2 %, *P* = 0.001) and dyslipidemia (73.7 % vs 34.6 %, *P* < 0.001) were more common in patients taking statins than in those who were not. Patients taking statins had significantly higher eGFR (47.3 ± 12.6 vs 42.0 ± 17.7 ml/min/1.73 m^2^, *P* = 0.001). Triglyceride levels were significantly higher in the patients taking statins (151.7 ± 111.0 vs 127.8 ± 79.1 mg/dl, *P* = 0.015). Patients taking statins more commonly used dual antiplatelet therapy (98.6 % vs 91.8 %, *P* = 0.001; Table 1).

**Table 1:** Patients’ characteristics

	Statin (−) (*n* = 182)	Statin (+) (*n* = 209)	*P* value
Age (years)	72.0 ± 9.9	68.7 ± 10.1	0.001
Male gender	139/182 (76.4)	163/209 (78.0)	0.704
Obesity	50/177 (28.2)	90/208 (43.3)	0.002
ACS	65/182 (35.7)	76/209 (36.4)	0.894
Prior MI	26/182 (14.3)	32/209 (15.3)	0.776
Prior PCI	16/182 (8.8)	31/209 (14.8)	0.067
Prior CABG	11/182 (6.0)	13/209 (6.2)	0.942
Hypertension	131/182 (72.0)	142/209 (67.9)	0.386
Diabetes mellitus	67/182 (36.8)	79/209 (37.8)	0.841
Dyslipidemia	63/182 (34.6)	154/209 (73.7)	<0.001
Hyperuricemia	88/182 (48.4)	76/209 (36.4)	0.017
Cigarette smoking	48/182 (26.4)	59/209 (28.2)	0.681
Family history	22/182 (12.1)	37/209 (17.7)	0.122
eGFR (ml/min/1.73 m^2^)	42.0 ± 17.7	47.3 ± 12.6	0.001
Total cholesterol (mg/dl)	185.3 ± 36.2	193.0 ± 45.9	0.066
LDL (mg/dl)	108.0 ± 32.3	113.0 ± 37.5	0.160
HDL (mg/dl)	51.4 ± 16.6	49.4 ± 13.3	0.197
TG (mg/dl)	127.8 ± 79.1	151.7 ± 111.0	0.015
Glucose (mg/dl) (JDS)	139.8 ± 65.4	137.6 ± 52.7	0.716
HbA1c (%)	6.0 ± 1.0	6.2 ± 1.3	0.113
LVEF (%)	58.7 ± 16.3	59.4 ± 14.5	0.688
DAPT	167/182 (91.8)	206/209 (98.6)	0.001
Anticoagulant therapy	21/182 (11.5)	19/209 (9.1)	0.426
β-Blockers	67/182 (36.8)	80/209 (38.3)	0.766
ACE-Is	25/182 (13.7)	43/209 (20.6)	0.075
ARBs	85/182 (46.7)	92/209 (44.0)	0.595
RAS-I	107/182 (58.8)	130/209 (62.2)	0.491
CCBs	83/182 (45.6)	96/209 (45.9)	0.948
Vasodilators	67/182 (36.8)	78/209 (37.3)	0.917
Antihyperuricemic	42/182 (23.1)	43/209 (20.6)	0.550
Diuretics	53/182 (29.1)	44/209 (21.1)	0.065
Aldosterone antagonist	22/182 (12.1)	16/209 (7.7)	0.140
Antidiabetic	36/182 (19.8)	52/209 (24.9)	0.228
Insulin	8/182 (4.4)	7/209 (3.3)	0.591
LMT	15/182 (8.2)	23/209 (11.0)	0.358
MVD	121/182 (66.5)	144/209 (68.9)	0.610
BMS	61/182 (33.5)	61/209 (29.2)	0.357
DES	103/182 (56.6)	146/209 (69.9)	0.007

Data are expressed as mean ± SD, or counts (percentage)

*ACS* acute coronary syndrome, *Prior MI* prior history of myocardial infarction, *Prior PCI* prior history of percutaneous coronary intervention, *Prior CABG* prior history of coronary artery bypass graft, *eGFR* estimated glomerular filtration rate, *LDL* low-density lipoprotein cholesterol, *HDL* high-density lipoprotein cholesterol, *TG* triglyceride, *JDS* Japan Diabetic Society, *HbA1c* hemoglobin A1c, *LVEF* left ventricular ejection fraction, *DAPT* dual antiplatelet therapy, *Statin* HMG-CoA inhibitor, *ACE-I* angiotensin-converting enzyme inhibitor, *ARB* angiotensin II receptor blocker, *RAS-I* renin–angiotensin system inhibitor, *CCB* calcium-channel blocker, *LMT* left main trunk disease, *MVD* multivessel disease, *BMS* bare-metal stent, *DES* drug-eluting stent

### Echocardiographic findings

Ultrasound cardiography showed that the left ventricular ejection fraction (LVEF) was comparable between patients taking and not taking statins (59.4 % ± 14.5 % vs 58.7 % ± 16.3 %, *P* = 0.688; Table 1).

### Angiographic findings

Coronary angiography showed that the prevalence of left main trunk (LMT) disease (11.0 % vs 8.2 %, *P* = 0.358) and multivessel disease (MVD) (68.9 % vs 66.5 %, *P* = 0.610) were comparable between patients using and not using statins (Table 1).

### Clinical outcomes

**Fig. 1:**
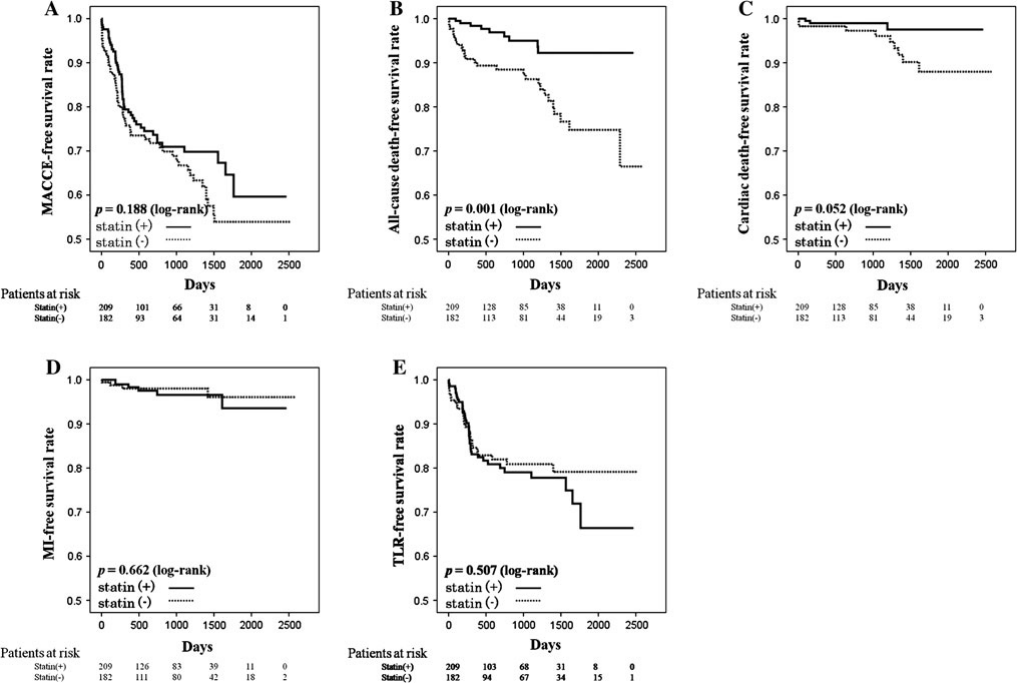
Kaplan–Meier curves for the major adverse cardiovascular and cerebrovascular event (*MACCE*)-free survival rate (**a**), all-cause death-free survival rate (**b**), cardiac death-free survival rate (**c**), myocardial infarction (MI)-free survival rate (**d**), and target lesion revascularization (TLR)-free survival rate (**e**)

All-cause death and cardiac death occurred in 9 and 3 patients taking statins, respectively, and 29 and 10 patients who were not taking statins (Table 2). Kaplan–Meier curves and the log-rank test revealed that the frequencies of MACCE (*P* = 0.188) and cardiac death (*P* = 0.052) tended to be lower in patients using statin therapy than in those who did not, and the frequency of all-cause death was significantly lower in patients taking statins than in those who did not (*P* = 0.001). The frequencies of MI (*P* = 0.662), and TLR (*P* = 0.507) were comparable between patients using and not using statins (Fig. 1).

**Table 2:** Clinical outcomes

	Statin (−) (*n* = 182)	Statin (+) (*n* = 209)	*P* value
MACCE	58/182 (31.9)	54/209 (25.8)	0.188
All-cause death	29/182 (15.9)	9/209 (4.3)	<0.001
Cardiac death	10/182 (5.5)	3/209 (1.4)	0.026
MI	4/182 (2.2)	6/209 (2.9)	0.674
TLR	28/182 (15.4)	40/209 (19.1)	0.329

Data are expressed as counts (percentage)

*MACCE* major adverse cardiovascular and cerebrovascular event, *MI* myocardial infarction, *TLR* target lesion revascularization

### Predictors of death

Univariate Cox regression analysis showed that obesity, acute coronary syndrome (ACS), eGFR, glucose levels, LVEF, statins, diuretics, and aldosterone antagonists were associated with death (from all causes) in CKD patients after PCI (Table 3). Multivariate Cox regression analysis, including the significant predictors above and the marginally significant ones (*P* < 0.10) (including age, hypertension, and dyslipidemia) in the univariate model, showed that low eGFR, poor LVEF, and the absence of statin treatment were independent predictors of death in CKD patients after PCI (Table 4).

**Table 3:** Unadjusted predictors for all-cause death

	*P* value	Hazard ratio	95 % CI
Age (years)	0.057	1.032	0.999–1.067
Male gender	0.508	0.784	0.380–1.614
Obesity	0.015	0.336	0.139–0.810
ACS	0.006	2.461	1.291–4.690
Prior MI	0.242	1.595	0.729–3.490
Prior PCI	0.688	1.213	0.473–3.115
Prior CABG	0.762	0.803	0.193–3.334
Hypertension	0.073	0.557	0.294–1.056
Diabetes mellitus	0.045	1.917	1.013–3.626
Dyslipidemia	0.072	0.553	0.290–1.053
Hyperuricemia	0.879	1.051	0.554–1.994
Cigarette smoking	0.113	0.515	0.227–1.171
Family history	0.144	0.415	0.128–1.351
eGFR	<0.001	0.963	0.947–0.978
Total cholesterol (mg/dl)	0.209	0.995	0.986–1.003
LDL (mg/dl)	0.119	0.992	0.982–1.002
HDL (mg/dl)	0.430	1.008	0.988–1.029
TG (mg/dl)	0.119	0.996	0.991–1.001
Glucose (mg/dl) (JDS)	<0.001	1.008	1.004–1.012
HbA1c (%)	0.216	1.164	0.915–1.480
LVEF (%)	<0.001	0.953	0.936–0.970
DAPT	0.847	1.152	0.274–4.851
Anticoagulant therapy	0.659	0.767	0.236–2.495
Statins	0.001	0.287	0.135–0.608
β-Blockers	0.728	1.122	0.585–2.151
ACE-Is	0.861	0.929	0.407–2.118
ARBs	0.115	0.568	0.281–1.148
RAS-I	0.127	0.608	0.321–1.152
CCBs	0.038	0.466	0.226–0.960
Vasodilators	0.550	0.815	0.416–1.594
Diuretics	0.006	2.461	1.290–4.693
Aldosterone antagonist	<0.001	3.840	1.856–7.943
Antidiabetic	0.850	1.075	0.509–2.271
Insulin	0.688	0.666	0.091–4.858
LMT	0.462	1.424	0.555–3.656
MVD	0.275	1.544	0.707–3.370

*ACS* acute coronary syndrome, *Prior MI* prior history of myocardial infarction, *Prior PCI* prior history of percutaneous coronary intervention, *Prior CABG* prior history of coronary artery bypass graft, *eGFR* estimated glomerular filtration rate, *LDL* low-density lipoprotein cholesterol, *HDL* high-density lipoprotein cholesterol, *TG* triglyceride, *JDS* Japan Diabetic Society, *HbA1c* hemoglobin A1c, *LVEF* left ventricular ejection fraction, *DAPT* dual antiplatelet therapy, *Statin* HMG-CoA inhibitor, *ACE-I* angiotensin-converting enzyme inhibitor, *ARB* angiotensin II receptor blocker, *RAS-I* rennin–angiotensin system inhibitor, *CCB* calcium-channel blocker, *LMT* left main trunk disease, *MVD* multivessel disease, *CI* confidence interval

**Table 4:** Adjusted determinants of all-cause death

	Univariate *P* value	*P* value	Hazard ratio	95 % CI
Age	0.057	0.172	1.028	0.988–1.069
Obesity	0.015	0.260	0.586	0.232–1.484
ACS	0.006	0.889	0.943	0.413–2.150
Hypertension	0.073	0.345	0.687	0.316–1.497
Diabetes mellitus	0.045	0.214	1.672	0.743–3.764
Dyslipidemia	0.072	0.568	1.268	0.560–2.871
eGFR	<0.001	0.011	0.973	0.952–0.994
Glucose	<0.001	0.259	1.003	0.998–1.007
LVEF	<0.001	0.005	0.963	0.939–0.989
Statin	0.001	0.023	0.348	0.140–0.866
CCBs	0.038	0.748	1.156	0.479–2.791
Diuretics	0.006	0.653	0.809	0.322–2.035
Aldosterone antagonists	<0.001	0.441	1.559	0.504–4.826

*ACS* acute coronary syndrome, *eGFR* estimated glomerular filtration rate, *LVEF* left ventricular ejection fraction *Statin* HMG-CoA inhibitor, *CCB* calcium-channel blocker

## Discussion

The present study showed that statin treatment reduced all-cause mortality of Japanese CAD patients after PCI complicated with CKD. Multivariate Cox regression analysis showed that the absence of statin treatment, as well as low eGFR and poor LVEF, was an independent predictor of all-cause death of CKD patients after PCI.

Statins are promising agents for CAD, as supported by a plethora of clinical evidence [7, 9, 11]. In Western countries, statins are administered to 80 %–90 % of CAD patients [26]. By contrast, in the present study CKD patients received statins after PCI in 54 % of cases. Administration of statin therapy was determined by the attending physician, based on the guidelines. Although statins were prescribed for patients, they were often not taken or discontinued for different reasons. These findings should reassure physicians that the use of statin treatment is critical for patients with CAD after PCI to prevent adverse outcomes. Because underuse of statins in patients with CKD may relate to potential toxicity concerns in patients with impaired renal function, the recommendation of the National Lipid Association Statins Safety Assessment Task Force [27] indicated that CKD should not preclude the use of statins.

It was controversial that treatment with statin might prevent future cardiac events of patients with renal dysfunction [28–31]. In the 4D study, atorvastatin had no statistically significant effect on the composite primary end point of cardiovascular death, nonfatal MI, and stroke in patients with diabetes receiving hemodialysis, suggesting that the benefit of statin treatment was limited when intervention with statins was postponed until patients had reached end-stage renal disease [32]. In this study, the number of patients receiving hemodialysis was only 8.7 % (34/391) and most of the study patients had mild CKD. Hence, early statin administration in CKD patients might provide clinical benefit. These findings should reassure physicians that the use of statin treatment is critical for patients with CAD after PCI in preventing adverse outcomes. There may thus be room for further improvement of clinical outcomes by increased use of statins.

To our knowledge, limited data have shown the effects of statin therapy for unselected Japanese CKD patients with CAD who underwent PCI with a broad range of lipid levels; the present study therefore newly demonstrates importance of statin treatment by revealing a significant reduction in all-cause mortality of CKD patients. However, we do not fully understand why the statin treatment reduced the all-cause mortality rate in patients with CKD and CAD who underwent PCI. Statins are known to have pleiotropic effects, including improving endothelial function [33], decreasing platelet activity [33], and attenuating inflammation [34, 35], which may contribute to the decreased mortality rate in CAD and CKD patients.

We found a discrepancy between all-cause mortality and adverse cardiac events in the present study. Previous studies reported that TLR and other cardiovascular events are reduced by statin treatment. This was not observed in the present study. The effect of statin treatment on plaque regression acts in a dose-dependent manner, which may underlie this discrepancy between the present study and previous ones conducted in Western countries. In addition, the lipid profiles of subjects in this study were almost within normal range. Because baseline levels of low-density lipoprotein cholesterol may alter the overall benefit of statins, it is possible that statins, used in doses approved for Japanese subjects, were not sufficient to reduce the restenosis rate or atherosclerotic burden during the follow-up period. Furthermore, PCI was performed in our institute using a careful stent-deployment technique with routine use of intravascular ultrasound. Although the pleiotropic effects of statins can contribute to reduced mortality, the reasons for the discrepancy between mortality and morbidity in our study are not fully explained. Further studies are required to investigate the mechanisms by which statin treatment may prevent fatal events.

### Study limitations

We recognize that the present study had several limitations. First, this study reflects the experience of a single center, and the number of patients in the study was small. The statistical power may not be sufficient for negative data to be conclusive. Accordingly, more studies in a larger cohort are needed. Second, we divided patients into two groups, a statin group and a nonstatin group, irrespective of statin types and doses. Further study will be needed to clarify the appropriate types and doses of statin treatment for CAD and CKD patients. Finally, cholesterol profiles and medications were not assessed during follow-up, and some patients in the nonstatin group may have subsequently begun to take statins, while others in the statin group may have discontinued the treatment. However, this misclassification bias would have led us to underestimate the true effects of statin treatment. Despite these limitations, we believe that our findings provide valuable information to inform future treatment for patients with CKD and CAD after PCI, because the target population is representative of patients seen in daily clinical practice and the statin doses in this study were consistent with those used in clinical settings in Japan.

## Conclusion

Statin treatment reduced all-cause mortality in Japanese CKD and CAD patients who underwent PCI. The absence of statin treatment, as well as poor left ventricular function and reduced renal function, was an independent predictor for all-cause death of CKD patients, suggesting a crucial role of statin treatment in long-term clinical outcomes of CKD and CAD patients.
